# Nonsurgical Treatment of the Commonest Form of Deafness1Read before the Section on Ophthalmology, Otology, Laryngology and Rhinology, Buffalo Academy of Medicine, October 14, 1901, and Park Medical Club.

**Published:** 1901-12

**Authors:** George F. Cott

**Affiliations:** Buffalo, N. Y., Clinical Professor of otology, University of Buffalo.


### Nonsurgical Treatment of the Commonest Form of Deafness.[1]

By GEORGE F. COTT, M. D., Buffalo, N. Y.,
Clinical Professor of otology, University of Buffalo.

IN TAKING up this subject I am mindful of the difficulties to be overcome. To treat catarrhal deafness successfully, it first becomes necessary to make a correct diagnosis. We all have had some experience in treating deafness. I may sum it up in one word: politzerisation. I make no distinction in the kind of deafness; all are treated in nearly the same way. In this paper I wish to point out the fallacy of the present method. Politzerisation is a great remedy when properly applied in the right case. Before instituting treatment, however, let us examine our patient and ascertain with what kind of deafness he or she is afflicted.

Case I.—We have here a little patient, aged 4, whose mother states that she cannot hear unless addressed in a loud voice. Deafness has been noticed for a year past. The child takes cold easily and often, during damp weather, coughs at night, without awaking. Often restless during sleep. We examine the ears externally and find them normal, then the canal and the drum-head, commonly referred to as the ear drum and find those also quite normal. Child may be fat or lymphatic, or thin and poorly nourished, or its general appearance good. We examine the nose and find often thick secretions which the child does not seem able to dislodge. Next, the throat is inspected and moderately hypertrophied tonsils are found. Then upon examining the post-nasal space, we notice a mass of tissue which looks like the tonsil lower down, and we at once make our diagnosis of deafness, partially due to the irritation caused by the excessive amount of lymph tissue behind the nasal cavity, and partially by closure of the eustachian tube by the same. In this case removal of the upper tonsil generally improves hearing, but occasionally other treatment must be instituted according to the needs of the individual case, such as tonics, daily cod liver oil baths, fresh air, sunlight, proper food, and the like. During this time politzerisation will aid the improvement very much and since the eustachian tubes in children are more or less patulous, the politzer bag may be used with advantage by simply pressing the balloon without the child swallowing and the air readily enters the middle-ear. In a very short time the child hears as good as ever.

Case II.—A boy, aged 12, well developed, apparently perfectly healthy, but cannot hear except when spoken to very loudly. He never hears ordinary conversation. Has been

---

1. Read before the Section on Ophthalmology, Otology, Laryngology and Rhinology, Buffalo Academy of Medicine, October 14, 1901, and Park Medical Club.

treated a number of months by a certain doctor and again by another. What was the treatment? The answer is, air blown into the nose while the boy was swallowing water. No result. Anything else? No. Ears appear quite normal. Cause must be somewhere else. Nose shows not sufficient cause, throat not very bad, post-nasal space, large pharyngeal tonsil not, however, covering the openings of the eustachian tube, but yet may be causing congestion of the mucous membrane lining the tubes and the middle-ear. Adenoids removed, boy hears but little better. Next day throat washed out with a mild non-irritating alkaline wash and then the tubes are inflated, gently, of course; perhaps 15 or 20 lbs. pressure; this is repeated every other day and in a few days to several weeks hearing is perfectly restored.

Case III.—Young man, aged 25, deaf for eight years, does not remember cause. Treated by several doctors, including advertising specialists, and using patent ear drums. All doctors treated him alike: politzerisation. No improvement whatever. Why not? Patient is rather young for sclerosis, no history of zymotic disease, never any discharge from the ear. Rinnes's test negative; Weber's positive. Drumhead somewhat thick and opaque, cone of light absent. Nose shows inferior hypertrophies, nothing more; catches nasal cold very easily. Loud voice heard four feet, whisper not at all; accumeter, two inches; watch only upon contact. Diagnosis: catarrhal deafness due to hypertrophy. Politzerisation negative. Evidently some other measures must be adopted. Nasal hypertrophies are first reduced and the susceptible mucous membrane brought under subjection. Catheter then used; 25 or 30 lbs. pressure brought to bear and patient at once hears the watch 4 inches, and loud voice 8 feet. Prognosis: good.

Case IV.—Man, aged 55, carpenter, deaf for number of years; no known cause. Heart sounds weak, for which strychnine was given; occasional tinnitus; drumhead thickened and some retraction of malleus. Politzerization negative. Nose and throat normal. Catheter gave but slight result, yet enough to warrant treatment. Prognosis: only fair. Diagnosis: old hypertrophy merging probably into sclerosis, which age and length of trouble would indicate. Bougie passed through catheter twice a week, allowing it to remain ten minutes. After several weeks, 30 lbs. pressure through catheter improves hearing perceptibly. After several months politzer air bag opens tubes fairly well and patient hears clock tick in his bed-room, which he said he was never able to hear before.

Case V.—Woman, aged 48, well nourished, yet complaining of all sorts of trouble which her physician properly diagnosticated as incident to the menopause, through which period she was just passing. Bowels constipated, headache, backache, noises constantly in her head, causing loss of sleep, conjunctivæ not sensitive, and different areas of skin anesthetic. Deafness progressive for a number of years. Drumhead retracted, malleus drawn in a horizontal position, lower border sharpely outlined. Could hear very well when riding in street car. Tubes easily inflated and eustachian cushion pale. No improvement after inflation. Rennes's test positive; Weber's indifferent. Diagnosis: sclerosis. Prognosis: bad, but amelioration of symptoms possible. Ears not treated at all. Was given ovarian extract, 10 grains, three times a day; bowels regulated and daily spinal douche with cold water ordered, preferably in the morning when the patient slept fairly well, or at night when the opposite is the case. Report in two weeks. Patient much better in general health; noise in ears sometimes entirely gone and when it returns is much milder. Hearing improved because tinnitus is lessened. Subjective sounds relieved greatly by tr. cimicifuga, 10 drops, three times a day.

Case VI.—Female, aged 25, fairly well nourished, but markedly hysterical. Deafness ten years; no apparent cause. Examination indicated hypertrophy of mucous membrane of middle-ear and tubes. Tubes difficult to open with catheter. "Catches cold all the time," as she puts it, and often sore throat, worries a great deal over domestic troubles. Prognosis: fair considering length of time of trouble. Nasal mucous membrane treated first, and several weeks later tubes were inflated through catheter, with considerable difficulty. After several weeks more this became perceptibly easier, but improvement in hearing lasted but a short time, when it became as bad as ever. Turned her over to family physician and ordered all treatment to ears stopped until hysterical condition had improved. A year later patient called again. Hysteria all gone and she was joyful and happy. Although no treatment was given for deafness, hearing had so much improved that she could converse with but slightly louder voice than ordinary conversation.

### CASES COMMONLY NEGLECTED.

Such are the average cases which come under our observation from time to time. Although those quoted above are hypothetical, yet they are typical of the average. Any number of partially deaf people are walking our streets daily who had undergone treatment for months or years, principally by the advertising specialists and whose pictures you may see in the daily papers as typical examples of persons cured by three months' treatment, the first thirty days free of charge.

Catarrhal deafness of the kind referred to in this paper and which is so often improved by proper treatment, is just the kind of condition very much neglected. Physicians in general expect too much in too short a time. Let us make a differential diagnosis of the average case and follow certain rules in conducting the treatment while giving a guarded prognosis.

### ASCERTAIN CAUSE OF DEAFNESS.

First, what is the cause of deafness, chronic catarrh of the hypertrophic or sclerotic variety? Is it due to zymotic disease or, if profound, is it due to stapedial anchylosis? Chronic discharge, or has the discharge stopped? Is the drumhead perforated, large or small, and if so may it be benefited by an artificial drumhead? Is it due to malaria or rheumatism? Is it feigned? Did it come on suddenly or was it first noticed when waking? Is there constant noise in the ears or only at times? Does the patient hear better in a noise or does the hearing then become worse? Is the deafness due to labyrinthine disease, or perhaps of central origin? Perhaps the patient can hear but not be able to repeat the words—amnesic aphasia. Did it follow a box on the ear? Is deafness double or affecting one ear only? Is patient worse in damp weather? Are other members of the family deaf? Is patient a deaf mute? Is he old, young, or middle aged? All these questions are of more or less importance.

### DIFFERENTIAL DIAGNOSIS.

In differentiating between common catarrhal and adhesive or the sclerotic variety, certain symptoms must be taken into consideration. Adhesive catarrh occurs in later life, 86 per cent., while the other is an affection of the young; 20 per cent. of all ear diseases are of this class. Nor is it so slow in its progress. While the one may be years in developing serious deafness, the other can usually be referred to the time of its onset. The one grows progressively worse with or without treatment, while the other is generally markedly benefited and often within a very short time. Paracusis is prominent in the sclerotic and usually permanent, while it is comparatively rare in the hypertrophic catarrhal. Subjective noises are greatly diminished in the one and seldom influenced by ordinary treatment in the other. In a certain percentage of cases differential diagnosis is not so clearly made and may require treatment for some time before giving a final prognosis. In fact, many things must be considered before a conclusion is reached.

### PROGNOSIS.

After having decided that our patient has the commonest kind of deafness, known as hypertrophic catarrhal, our prognosis is, with proper treatment, fairly good; that is, the patient may be improved materially. Now we assume that we have put the nose and throat and post nasal space in normal condition, the habit of catching cold has been stopped, the condition known as aprosexia, that is, a proper want of concentration of mind upon any subject for any length of time, relieved; hawking, hemming, spitting and dropping down the throat checked; we then ascertain the degree of deafness and its immediate improvement after treatment. The politzer bag, we assume, does not remedy matters much. The catheter is used and found to open the tubes quite readily, which we can tell by using the auscultative tube. The watch is then heard several inches, whereas before treatment it could only be heard on contact.

This treatment alone may have to be kept up for several weeks or months, then the air bag may be substituted. In the meantime patient must be tested by general conversation, using only familiar terms and it will be noticed that patient's eyes are watching the speaker's lips. At first it is well to have the ear turned toward the speaker, and when the habit has been overcome, the patient should be instructed to look into the eyes of the physician and never on the lips. Now you may test the hearing power from time to time, but be careful to use only familiar words or you will be led astray, as an unfamiliar sound will never be understood at this stage. The patient will hear the words but cannot repeat them. Why? Remember the patient has been deaf a number of years and forgotten a great many sounds familiar to you. It becomes necessary to teach the auditory nerve anew what these lost sounds are, and that takes a long time.

A patient will come for treatment occasionally, after several months of labor on your part, and exclaim that she is as bad as ever, that she was in company with some ladies and gentlemen last night and couldn't hear a word. Who was her company? Some strangers from out of town. That accounts for it. Sounds by strange people are unfamiliar to the auditory nerve. Yet my voice is very quickly appreciated, talking no louder than before. This is important to remember or both will be discouraged. It takes usually many months to make perceptible improvement, and yet in the majority of cases improvement follows even general constitutional treatment and the tubes gradually open. The younger the patient the better the result in the shorter time. After middle age, prognosis must be more guarded and very much so when heredity plays a part in deafness. In a certain number of cases the patient may be given a rest for several months and treatment begun again, at which time it will be noticed that marked improvement has taken place.

METHOD OF TREATMENT.

It also happens that patients will claim no improvement whatever after the rest. By consulting your record blank and comparing the present condition with the last year, you may find 100 per cent. improvement not apparently perceptible to the patient until told of it. Treatment with variations may have to be carried on from several months to several years. Perhaps by catheter, then air bag, then rest, then air bag again, teaching familiar sounds which were lost by lip reading, and the like.

This latter method ought to be considered of great importance. Patients who have been deaf for a short time, or who were along in years or approaching middle age, will not need so thorough a course of instruction as younger ones. The average patient is a good way this side of forty and therefore a general rule may cover the average case. After patient has been brought to the condition when we begin to treat the ears, confidence is quite thoroughly established. Lip-reading is strictly interdicted, because patient has formed the habit to substitute the one for the other and never hears more than some kind of sound, regardless of what you say, or the pitch you employ, and consequently hears nothing, but endeavors to read the motions of the lips.

I find, after a short time, the patient will ask no more repetition of sentences. After several months of this precaution, the physician will be informed his voice is heard quite distinctly, but other people cannot be heard any better than before. Here is another problem to be solved; but so far I have proved that hearing has materially improved when compared with the condition of the first visit. My voice is distinctly heard because its sound has become familiar. The same tactics must be employed with other people who converse with patient. It is far better to have a sentence repeated several times than resoit again to lip reading, only the patient expects too much after some improvement has taken place and is often ungrateful for not receiving more than nature will allow. It is well at first to have the ear turned towards the speaker so as again to familiarise the auditory nerve and brain center with the sound long forgotten, and this sound passing into the ear directly is better appreciated than when the sound waves make an acute angle. In a little while, however, the patient may be instructed to face the sound, for "out of the eye speaketh the soul," and common sentences in daily use will be distinctly understood. In this way persons who could never hear a sermon can sit ten or twenty feet away from the preacher and catch most of the discourse. This method may occupy the better part of years, but will succeed if proper care be taken and neither physician nor patient tire of the treatment.

### PATIENCE, PATIENCE.

It was a habit formerly to give up in despair if a patient did not improve within a few weeks, or after that time the patient would remain away voluntarily. This ought to be discouraged. If no hope is held out, it is quite natural to lose faith and try quacking, which at all events promises something, and sometimes a great deal, but it is never accomplished. We have it in our power, even though we promise very little, to accomplish a great deal. You may talk French, German or Latin, it's all the same to the catarrhal deaf, but if you repeat a sentence from any language often enough, patient will distinctly hear and differentiate between pitch and variations of sound. What seemed all Greek, will finally be woven into beautiful sounds and often provoke a smile of recognition. These people must learn their language a second time. The auditory nerve lies dormant, and the brain center unimpressionable and therefore must be again taught as in a child. This was forcibly brought to my mind by Professor Urbantschitsch at the Vienna Poliklinic, who made the discovery that deaf mutes could hear many sounds but had never been taught their significance. He has written several papers on the subject since. He would let the patient know what the object in his hand was. and then repeat the name in a loud voice several inches from the ear, and often after several lessons it would be pointed out after having heard the sound, thus conclusively proving that sound did exist for those unfortunates and only needed further patient development.

### GOOD CONDITION NECESSARY.

All this time the general condition must not be lost sight of; urine examined, bowels kept open, regular bathing and sleep, avoiding overexertion, prevent worry and the like. Patients treated in this way, the internal ear being normal, will gradually improve. Even sclerosis, when not gone too far, will be benefited. When, however, anchylosis of the stapes exists, prognosis is always bad. Hearing better in a noise is usually an accompaniment of sclerosis and should put one on guard. Past middle life hearing often decreases, which must be borne in mind.

### TREATMENT BY AIR BAG.

Catarrhal deafness in younger people is treated by all practitioners with the air bag, its object being to open the Eustachian tube and by that treatment keep it permanently open. Why? When the tubes are patulous the pressure within the middle-ear is equalised as in normal hearing. When the tubes are closed, the air contained in the drum is gradually absorbed and the pressure from without causes the drumhead to be pushed inwards, and thus we often see the deformity called retraction. The action of compressed air is felt in the Eustachian tubes, the membrana tympani and the structures covering the fenestræ ovalis and rotunda. The tube is distended, secretion removed and reduction of hyperemia favored, it forces the drumhead outwards and thus drawing with it the ossicles, which, if formerly much impeded, will immediately react to vibratory motion. In acute cases with severe pain, mild inflation will often relieve the pain instantly. When fluid is present without pain, it is not safe nor recommended to inflate the ears.

Bands of connective tissue, which tie down the ossicles, can often be influenced by forcible stretching, in crowding the membrana tympani outwards. In this way abnormal labyrinthine tension may be relieved by lessening stapedial pressure. In short, in reëstablishing normal air pressure we favor subsidence of hyperemia, hyperplasia, or even true thickening by gradual absorption, which can be noticed by improved hearing and which in true hypertrophy is very slow.

### SCHOOL CHILDREN.

Probably all of our teachers in the different schools have noticed some of their pupils not so attentive as others, or duller intellectually than the average child. If the children were properly examined it would be found that in many cases there is a definite degree of deafness, which lies at the bottom of the trouble. A vast majority of these cases might be relieved and the children become as active as the rest. It may be well to bear in mind that the younger the patient the better the prognosis, and to the family physician is delegated the task of properly directing his patients, and getting thereby everlasting gratitude.

In closing, permit me to remark, as soon as the laity are educated to the fact that hearing can be restored in a great measure, by proper treatment, extending over a sufficient period of time, there will not be nearly as much deafness as there is at present.

531 Mooney-Brisbane Building.

